# Time Series Analysis for Spatial Node Selection in Environment Monitoring Sensor Networks

**DOI:** 10.3390/s18010011

**Published:** 2017-12-22

**Authors:** Siddhartha Bhandari, Neil Bergmann, Raja Jurdak, Branislav Kusy

**Affiliations:** 1 School of ITEE, University of Queensland, Brisbane 4072, Australia; siddhartha.raj.bhandari@gmail.com; 2 CSIRO/Data61, Pullenvale 4069, Australia; Raja.Jurdak@csiro.au (R.J.); brano.kusy@csiro.au (B.K.)

**Keywords:** wireless sensor networks, time series analysis, spatio-temporal analysis, environmental monitoring

## Abstract

Wireless sensor networks are widely used in environmental monitoring. The number of sensor nodes to be deployed will vary depending on the desired spatio-temporal resolution. Selecting an optimal number, position and sampling rate for an array of sensor nodes in environmental monitoring is a challenging question. Most of the current solutions are either theoretical or simulation-based where the problems are tackled using random field theory, computational geometry or computer simulations, limiting their specificity to a given sensor deployment. Using an empirical dataset from a mine rehabilitation monitoring sensor network, this work proposes a data-driven approach where co-integrated time series analysis is used to select the number of sensors from a short-term deployment of a larger set of potential node positions. Analyses conducted on temperature time series show 75% of sensors are co-integrated. Using only 25% of the original nodes can generate a complete dataset within a 0.5 °C average error bound. Our data-driven approach to sensor position selection is applicable for spatiotemporal monitoring of spatially correlated environmental parameters to minimize deployment cost without compromising data resolution.

## 1. Introduction

Environmental phenomena such as temperature, pressure, humidity, and soil moisture are dynamic processes. Understanding the spatio-temporal behaviour of these processes is relevant for understanding the surrounding ecosystem’s state. Environmental phenomena in general vary at a small spatio-temporal scale [1,2] that impact the local ecosystem. The microclimate (temperature, solar radiation and other phenomena at small scale) affects ecological changes in forests [3], soil characteristics in mine rehabilitation [4], and diseases in agriculture [5]. Thus it is crucial for many application scenarios to monitor environmental phenomena at high spatio-temporal resolution.

Understanding the spatio-temporal behaviour of the environmental phenomena requires development of an effective monitoring system. In past decades, weather stations have been the widely used for monitoring. However, weather stations are spatially sparse, and they only capture coarse-grained environmental variations, which are not sufficient for monitoring variations in small scale ecological processes.

Recently, wireless sensor networks have been widely used in small scale environmental monitoring as they can be economically deployed for fine-grained environmental sensing and monitoring. Example applications include city centre heat monitoring [2], air quality monitoring [6], building environment monitoring [7], soil moisture measurement [8], volcano monitoring [9], ocean exploration [10], and harsh mountain environment monitoring [11]. In most of these sensor network deployments, the number and positions of sensor nodes are selected based on intuition, domain knowledge, or cost constraints. There is currently a lack of an objective method for determining the best number of nodes and their spatial distribution. The challenge is that the optimal node number and locations are dependent on the specific spatiotemporal processes in the monitored environment. The dynamics of these processes are not known a priori, which is in fact the motivation for monitoring the environment. Two of the sensor networks deployed by our research lab for rainforest monitoring [12,13] and mine rehabilitation monitoring [4] are clear examples where the number of nodes that were deployed was not based on any evidence-based understanding of the number that would be needed. The question of the optimal number and placement of sensor nodes needed for adequate environmental monitoring remains a challenge, and that is the topic that this paper addresses.

In a real application scenario, it is important to know the optimal number of sensor nodes to be deployed and the best position to achieve the project’s scientific or business objectives. A large number of sensors incurs high deployment and operational costs. On the other hand fewer sensors may fail to capture sufficient local details. The design goal should be to achieve the scientific objectives at the most economical cost.

Strategies for determining the target number of deployment nodes vary from analytical to simulation-based approaches. Some of the strategies are theoretically-based where environmental phenomena are modelled as spatio-temporally correlated processes and suitable sampling strategies are developed, such as in [14,15] where Gaussian process modelling is used. In [16], Monte-Carlo simulation has been used to find the locations of nodes in space that produces the lowest spatial variability. In [17], a geometrical approach is used treating sensor deployment as an area coverage problem. Our approach balances theory with initial experimental evaluation of the sensor deployment area to ensure that the coverage is adequate for the specific deployment scenario.

This work considers a practical application scenario, using the example of a mine rehabilitation monitoring program over an area of several square kilometres [4]. The objective is to monitor small scale spatio-temporal variations using empirical data from a short-term, high density deployment to optimize the deployment of a number of long-term sensor nodes. First, a larger number of static sensor nodes are deployed across the sensor area. The observations at each sensor location form a time series while observation at different locations form multiple time series. A time series analysis framework is then applied on each individual series as well as at the multiple series. Co-integration analysis is then used to determine the relationships between series. Co-integration provides information on which time series are most similar to each other. Similar time series are used to determine one location that can be used as an estimate for its co-integrated locations. Redundant sensors can be re-used elsewhere, or alternatively initial deployments can be with a large number of low-cost, short lifetime sensors that are replaced by fewer yet more robust long-term sensors. Implementing our proposed co-integrated multiple time series analyses for temperature measurement in the mine rehabilitation scenario showed that 75% of the existing sensors are found to be co-integrated with the other 25%. In other words, similar temperature monitoring accuracy could be achieved with only 25% of the existing deployment. The proposed approach is general enough that it can be utilized in any spatio-temporal monitoring application.

The rest of the paper is organized as follows: Section 2 reviews previous work. Background information on the techniques used is described in Section 3. The analytical approach that is used and the algorithms developed for the approach are discussed in Section 4. Section 5 presents analytical results from the particular mine rehabilitation sensor network. Section 6 concludes the paper.

## 2. Previous Work

In [3], authors have described the association between ecological processes and microclimate (temperature, solar radiation and other phenomena at small scale). Temperature variation up to 8 °C within a small forest patch was reported and linked to ecological changes. The effect of small scale climatological condition on the development of a fungal disease on a potato crop and forest canopy was observed in [5,18]. Variation of temperature within a small urban area has been reported in [2] while the microclimate effects on soil characteristics in mine rehabilitation were reported in our previous work [4]. In all scenarios, variations in the environmental phenomena at small scale are observed and linked to environmental changes, motivating the need for accurate understanding of local microclimate conditions in many scenarios.

Environmental monitoring has a long history. As described in [19], The Australian Bureau of Meteorology has been monitoring climatic variables including temperature, pressure, sun radiation, and rainfall since 1957. However, only 4600 monitoring stations are installed to cover the whole 7.7 million square kilometres of Australia since the capital and operating costs of weather stations are very high [19]. Such a coarse-grained spatio-temporal environmental monitoring would not suffice for the small scale environmental impact analyses needed in mine rehabilitation [4] or rain forest monitoring [12] scenarios.

Significant research has been undertaken in the design of monitoring networks in sensor network applications. In general these works can be divided into three groups: mathematical, geometrical and simulation approaches. A selection is reviewed here. 

Environmental phenomena are modelled mathematically as a spatio-temporal random field where the monitoring network design problem becomes the problem of sampling the assumed random field. In [14,15], the phenomenon is modelled as a Gaussian process and sampling strategies are designed. In [15], the authors also deployed sensor nodes for some time to learn the parameters of the Gaussian process.

Another approach to design a sampling strategy has been the geometry-based approach. Within a spatial region various geometrical approaches are used to select the positions of the sensors. Voronoi tessellation, Delaunay triangulation, and cell declustering are some of the examples of these geometric arrangements [15]. In [17], Voronoi tessellation is used to optimize the node positions. The main issue with such approaches is the strong assumption regarding the nature of the process. Environmental phenomena will not have convenient geometrical regions of similarity. The limitation of such an approach in monitoring temperature is shown empirically in [15] where temperature variations among equidistant points are different.

Other work by Chen et al. [20] also addresses geographic sensor node selection, although in their case they select a subset of nodes from a heterogeneous collection of web-connected sensors for a particular application using a web-services approach. In their case geographical sensor selection is based on proximity and they do not provide a method for interpolating between sensor positions, which is the focus of this work. Wang et al. [21] have described a wide area technique for selecting the site of ground precipitation sensors to complement satellite observations. Their work is based on maximizing the geographical coverage of sensors, sensitive to local terrain conditions. Such techniques could be useful for determining the initial dense deployment of sensors, and is complementary to our work which then identifies the best subset of those sensor locations.

In the simulation approach, sensors are placed at selected points and simulated sample measurements are drawn from the expected sensor responses to check the quality of measurement. In [16], Monte Carlo simulation is used to choose sensor locations. However, this requires the spatio-temporal variability of the data to be estimated before any measurements are made.

Several studies have conducted time-series analysis in sensor networks [22,23,24,25]. Some works are based on simulation while others are based on real observed series. One common objective of all the studies has been to identify the nature of the time series from each sensor node and somehow use the knowledge to reduce communication among sensor nodes which is important in energy saving in resource constrained nodes. For example, in [24] sensor data is only transmitted when it cannot be accurately forecast by a time series model of past data. Most works are based on univariate analysis of measurements at one point. Our work considers the correlation of time series across space basing the analysis on multivariate or multiple time series. The main focus of our work is to explore co-integrated time series and exploit their behaviour to optimize the number of sensors needed to monitor the desired environmental phenomena at the required accuracy.

## 3. Background Information

This section briefly describes some background information required for this research. It includes information on time-series analysis and a technical specification of the environmental sensor network involved in this paper. Mathematical details are kept to a minimum, and readers are referred to [26] for further information.

### 3.1. Theory of Time Series Analysis

Time series analysis is a framework for analysing sequentially observed data in time. It involves analysing temporal correlation of the observation which can be used for identification of the process model that generates the data. Identification of the model helps in generalizing the nature of the underlying process and estimating past and future values based on available observations. Environmental phenomena that are observed sequentially at regular sampling intervals are best suited for this analysis. Environmental phenomena which form time series include temperature (T), solar radiation (S), soil moisture (M), and rainfall (R). Each variable has an observation at each sampling instant (t). The series of sampling intervals can be numbered (t_0_, t_1_, …, t*_n_*). The value of one variable at successive sampling instants forms a time series, e.g., (T_0_, T_1_, …, T*_n_*).

#### 3.1.1. Univariate and Multivariate Time Series

Univariate time series analysis is concerned with the study of a single time series. A series of temperature readings (T*_i_*) measured at one sensor node is an example of a univariate time series. Most of the environmental phenomena are measured in many locations generating multivariate time series which are correlated among themselves. Multivariate time series analysis is the process of analysing more than one time series at a time. Time series such as temperature (T_0_, T_1_, …, T*_n_*), solar radiation (S_0_, S_1_, …, S*_n_*), and soil moisture (SM_0_, SM_1_, …, SM*_n_*) have relationships between them that can be analysed under multivariate time series analysis. Similarly measurements of the same variable at different locations, e.g., temperature from different sensors, can be analysed using multivariate analysis.

#### 3.1.2. Stationary and Non-Stationary Time Series

A time series is called a stationary if it exhibits a consistent temporal statistical pattern. Such time series are amenable to time series analysis. If the moments of the time series such as mean and variance do not change with time, the series is called stationary to the mean and the variance. (M_0_, M_1_, …, M*_n_*) is called stationary of order (1, 2, 3, …, *n*) if moments (m_1_, m_2_, m_3_, …, m*_n_*) remain constant over time. For many applications, a time series is examined for second order stationarity. Second order stationarity is based on the assumption that the underlying phenomena is a Gaussian stochastic process for which first and second order moments (mean and variance) are sufficient to characterize it. A second order stationary time series whose covariance is such that Cov(Xt_1_, Xt_2_) can be generalized by Cov(τ) where τ = (t_1_ − t_2_) is called weakly stationary. Any time series that doesn’t show regularity about its moments is called a non-stationary time series, and simple time-series analysis techniques cannot be used. Temperature (T_0_, T_1_, …, T*_n_*) measured at a particular location is a good example of a non-stationary time series. Expected value, correlation, and variance all vary with time. Non-stationarity can occur due to seasonal variation, unknown noise involved or due to the nature of the underlying phenomena.

#### 3.1.3. Co-Integrated Time Series

Time series are called co-integrated if they show some similarity amongst themselves. If two time series are co-integrated, even if they are non-stationary, one can be estimated using the other. Many studies on co-integrated non-stationary time series have been conducted in the field of econometrics where various quantitative and qualitative economical series are analyzed [27,28]. Linear modelling can be performed among co-integrated series and ordinary least square estimation becomes the best unbiased estimation. Such estimation is mathematically tractable and statistically efficient. Most environmental phenomena are non-stationary in nature, so that linear estimation cannot be performed without the assumption of stationarity or some transformation. Assumptions may lead to invalid conclusions while some transformations render the data difficult to interpret in the transformed scale. If multiple time series exhibit co-integrated characteristics, no assumptions and transformation are needed. Co-integration analysis that has been proposed in econometrics for economic time series modelling is adapted for environmental time series in this work. As co-integration analyses search for similarly behaving series, this can help to determine environmental series which are redundant, and so the sensors generating those redundant time series are not needed.

#### 3.1.4. Augmented Dicky-Fuller Test

Before conducting any inferential analysis, the co-integrated nature of the time series needs to be validated. Researchers in [27,28] provided a framework to validate whether time series are co-integrated. The Augmented Dicky-Fuller (ADF) test is a statistical procedure that tests the stationarity hypothesis of a univariate time series. Given a time series, the ADF test fits varying degrees of autoregressive (AR) models and provides statistics needed for acceptance or the rejection of an initial non-stationarity hypothesis. Equation (1) shows an AR(1) process:

(1) $$y_{i}=c+\rho y_{i-1}+\varepsilon$$

where *ε* is a Gaussian white noise process with zero mean, and *c* is a drift constant.

The process is non-stationary if |*ρ*| ≥ 1 and the process is stationary if |*ρ*| < 1. In the ADF test, non-stationarity is tested for higher degrees of order p using Equation (2) i.e., to check if the time series fits an AR(*p*) model:

(2) $$\Delta y_{i}=\rho y_{i-1}+\overset{p-1}{\underset{j=1}{\sum }}b_{j}\Delta y_{i-j}+\varepsilon$$

where the difference operator ∆ is ∆*y_i−j_* = *y_i−j_* − *y*_*i−j*−1_.

The ADF test is available in the libraries of statistical computing platforms like R [29]. The Dickey-Fuller Test Statistic is a statistical measure that is used to confirm that the nodes are co-integrated. It should be less than a critical value determined by the number of observations, and the confidence of decision. The needed critical threshold value and related statistics for various orders of the process and the number of observations are tabulated in [27]. Table 1 below, shows the values for different numbers of observations and different confidence levels for an order 1 process. For a confidence level of 99% and more than 100 observations, it is common practice to choose a critical value of the ADF test statistic of −3.5.

### 3.2. Mine Rehabilitation Monitoring Sensor Network

**Figure 1:**
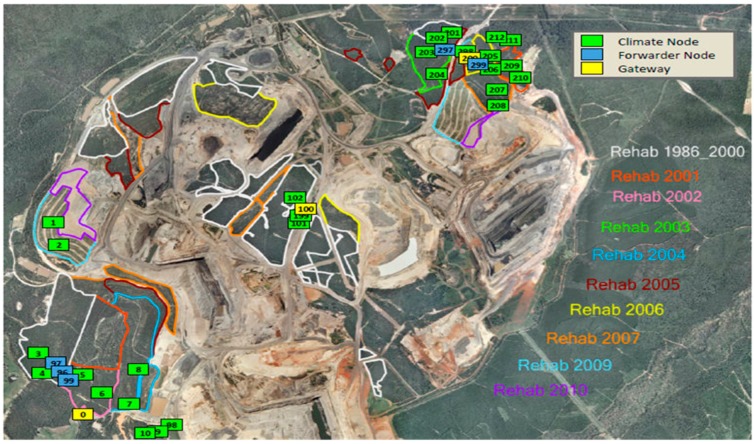
Meandu mine rehabilitation site and sensor deployment.

This study uses environmental sensor network data obtained from the Meandu open cut coal mine situated in a remote location of Queensland, Australia [4]. The industrial site of the mine is fairly large and spread across several sections of the mine site. The mine was established in the 1980s. Mining activity involves removing overburden, then removing the coal, and then replacing the overburden. After the mining is completed in one section, the rehabilitation phase commences. Rehabilitation involves restoring the previous environment, i.e., regenerating soil and re-establishing plants (grass, shrubs, trees) back to the condition of the natural environment. Sensor networks are deployed in rehabilitation sites, as shown in Figure 1, to monitor microclimate in order to assist with the timing of operations such as planting, and watering. Air temperature, soil temperature at two levels of depth, solar radiation, soil moisture, rainfall are measured in each rehabilitation site. The coloured outlines on the map show areas where rehabilitation has begun in different years from before 2000 up to 2010. The numbered boxes show the locations of sensor nodes.

The sensor network designed by CSIRO has been deployed in several rehabilitation sections. In the current deployment there are four sections, 12 sites and 24 transects in which 30 sensor platforms are deployed. For ground truth validation, several sophisticated weather stations are also deployed. Locations of the sensor nodes are selected based on the requirement of the rehabilitation monitoring. A custom sensor network platform using a 900 MHz IEEE 802.15.4 compatible radio was designed. A collection tree-based data collection protocol is used to for data communication from sensor to the gateway. The gateway station then forwards data to a centralized server using 3G connectivity. The server provides access to the data and further analysis. Technical details of the platform are given in [4].

### 3.3. Limitations and Assumptions

This paper represents a first exploration of using the time-series analysis method of co-integration for improved placement of sensors in an environmental sensing scenario. There are many assumptions and restrictions to the applicability of this model, as follows.

Firstly, the method is only applicable to sensing parameter fields that are spatially correlated, i.e., where values at locations that are close spatially tend to have similar values. Environmental parameters such as air temperature, humidity, wind speed and barometric pressure would be examples of such parameters. There are many parameters, especially in the built environment, which would not be amenable to such analysis, such as smart power meters in one street, or traffic density in nearby streets. Part of the analysis in the next section is to identify if time series data are suitable for this approach.

Another assumption is that spatial correlations between sensor readings persist over the long term. An initial exploration of the estimation error over a whole year based on one week of training data is presented in Section 5.4. 

In some situations, dense sensor deployments may be intended to detect data anomalies, for example a sudden increase in temperature due to an approaching forest fire. Again, since the approach here uses a few sensors to interpolate parameters at other locations, it will be less sensitive to local anomalies, and would not be suitable for such applications.

This initial investigation uses temperature as the example environmental variable, since it is easy to measure and changes relatively slowly. Our future work plans to extend this work to other sensors.

## 4. Proposed Analytical Methodology and Algorithms

### 4.1. Data Analytic Framework 

**Figure 2:**
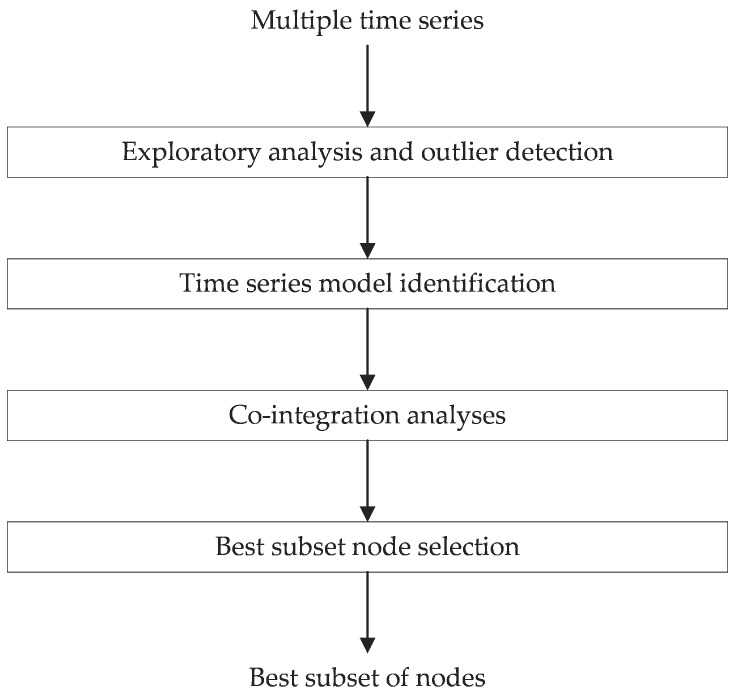
Multivariate time series analysis framework.

This section describes the analytical framework used for the analysis of the multivariate time series. Figure 2 shows the different steps involved in the analytical process. 

First, exploratory analysis of time series data looks for any significant inconsistencies. Spatially proximate sensors are plotted together for this. Outlier detection is performed including univariate and multivariate features. The detailed approach to performing outlier detection analysis is available in our previous work [4]. The next step is to identify the time series model. Stationary behavior of the series is analysed using an Augmented Dicky-Fuller test for each sensor. As expected, none of the periodic temperature time series are stationary. Co-integration analysis is then performed for all possible pairs of sensors. The result of the co-integration analysis is the confirmation or failure of the co-integration test of the pairs of the available sensors. After co-integration analysis, the Best Subset Node Selection step is performed that searches for the best possible subset of the sensor nodes that can estimate each of the time series.

### 4.2. Co-Integrated Series Selection Algorithm

Firstly, a decision must be made about which set of nodes are sufficiently close in location to be considered as possible co-integrated nodes. This means identifying a local neighbourhood of nodes. For example, in the experiments we describe here, 12 nodes in the north-east corner of the mine site (numbered 201 to 212 in Figure 1 above) are selected. They are within 1 km of each other. It would be less likely that nodes in the south-west corner of the mine would be as closely correlated. Within this neighbourhood, all possible pairs of nodes are examined.

The co-integrated series selection algorithm searches for the best co-integrated node for each sensor node. This algorithm starts fitting a linear model on one node with all the other nodes. After fitting the model each residual series is then evaluated for stationarity using the Augmented Dicky Fuller test. At the end of the run, the algorithm generates the best co-integrated node for each sensor node. 

In the case where the most correlated node has a Dickey Fuller test statistic which is above the critical value of −3.5, then it cannot be estimated accurately from other nodes, and that node would be one of the critical locations for a permanent sensor node.
**Algorithm 1:** Co-integrated time series selection**1: TS**
**←**
**sensor series****2: for each time series i do****3:   # fit a linear model with each other node j****4:   lm[i][j]←**
**linear model TS(i, j)**
**5:   resd[i][j]**
**←**
**residual(lm[i][j])****6: end for****7: for each residual i,j do****8:   # run Dicky − Fuller test****9:   DF [i][j]**
**←**
**ADFtest(resd(i, j))****10: end for****11: for each time series i do****12:   ts**
**←**
**maximum(abs(DF(i, j)))****13:   Cointegrated[i]**
**←**
**ts****14: end for**

### 4.3. Best Subset Sensor Nodes Selection Algorithm

After validating that the observed time series are co-integrated, a best subset nodes selection algorithm searches for the best subset of nodes that can be used to estimate the value at each unobserved location. At each location, the proposed algorithm starts searching for the best linear combination of observations at other locations that can reproduce the observed value. It is possible to set the maximum number of nodes to be searched from 1 to *N*, where *N* is the total number of available nodes. If the maximum node to be selected is set to 1, the algorithm selects a single best node for the estimation. The searching involves all available series. A linear combination of temperature at a particular location is calculated based on Equation (3):

(3) Y=βX+ε

where *β* = *(β*_0_, *β*_1_, …, *β_N_*) are corresponding linear weights and X is the matrix of variables with each column representing a single series. 

The least square cost function to minimize is given by *(Y* − *βX)^T^(Y* − *βX)* which when differentiated with *(β*_0_, *β*_1_, …, *β_N_*) provides the least squares unbiased estimation of the parameters as given by Equation (4):

(4) $$\hat{\beta }={\left(X^{T}X\right)}^{-1}X^{T}Y$$

In each iteration, the algorithm selects one more co-integrated series that has not been previously selected. The selection is based on the node whose addition to the subset most reduces the estimation error. After parameter estimation, the estimated value of this series based on the linear combination of other series can then be calculated for a test set (different from that used to select parameters) using parameters from Equation (4). 

In each iteration, the algorithm produces the training error for each series. Observing training errors, a suitable number of nodes can be selected which can generate all the series. This suitable number may be determined by operational requirements, e.g., one might have only 4 permanent sensing stations for deployment, and wish to choose the best four locations. Alternatively, this number could be chosen by scientific requirements, such as needing a maximum of 0.5 °C RMSE error at all the estimated positions. Finally, the number could be chosen on a statistical basis, such as identifying when adding an additional node does not significantly reduce the RMSE of estimated readings (using something like the heuristic “elbow” criterion in a graph of RMSE versus number of nodes). Pseudocode of the algorithm that selects the best subsets is given in Algorithm 2.
**Algorithm 2:** Best subset selection of *M* co-integrated nodes from *N* − 1 candidates for each of *N* nodes**1: # Search for the best subset of M sensors for each individual sensor, i****2: M**
**←**
**number of sensors in the subset****3: for each sensor i do****4:    searchspace**
**←**
**set of all sensors minus sensor i****5:    bestsubset[i]**
**←**
**NULL****6:    for j = 1 to M do {add one more sensor to best subset for i}****7:     lowest estimation error**
**←**
**infinity****8:     for each sensor k in searchspace****9:      fit linear model to sensor i using (k + bestsubset[i])****10:      if estimation error from linear model < lowest estimation error****11:       lowest estimation error**
**←**
**estimation error from linear model****12:       bestsensor**
**←**
**k;****13:      end if****14:     end for****15:     searchspace**
**←**
**searchspace**
**−**
**bestsensor****16:     bestsubset[i]**
**←**
**bestsubset[i] + bestsensor****17:    end for****18: end for**

It is useful to estimate the computational complexity of Algorithm 1 and Algorithm 2. Both algorithms basically have the same structure, which is for every pair of nodes, find a least squares estimator for one node from the other, and then calculate the goodness of fit, either by calculating the Dickey-Fuller statistic or the estimation error. The parameters which affect which affect computational complexity are N, the number of nodes, M the size of the best subset, C = 2M, the number of parameters that have to be estimated in the linear model, and S, the number of samples.

Equation (4) is the basis of fitting a linear model, and in terms of time complexity it consists of a matrix multiplication *X^T^X* which is O(C^2^S), a matrix multiplication *X^T^Y* which is O(CS) a matrix inverse which is order (C^3^), and a final matrix multiply which is O(C^2^). The calculation of the error metric or statistic consists of estimating S values from C parameters, O(CS). For the case where M = 1 (using just one estimator node), and therefore C = 2 is a constant, the order of one linear fit is O(S). If this is repeated for every pair of nodes, the total complexity is O(N^2^S). The N^2^ term suggests that it may be infeasible to apply this method directly to thousands of nodes, instead these nodes should be divided into disjoint neighbourhoods of less than 100 nodes. For M > 1 (i.e., larger subsets of estimators), the complexity grows to O(N^2^M^2^S), and so for these experiments we just use M = 1 to reduce the computation time. 

## 5. Analysis of Results

This section provides results obtained from implementing the proposed algorithms on the 12 sensors in a 1 km × 1 km area in the north-east of the Meandu mine site, as shown in Figure 1. The average distance between neighbouring nodes is about 100 m. Three weeks of temperature time series starting from 1 January 2013 are used for the analyses. The first week of data is used to select three “permanent” nodes from the 12, and to train models to estimate the other nine. Then the temperature is estimated at the nine positions from the three “permanent” nodes for 10 days, and the estimated temperature compared to the actual temperature at those nine positions. Temperature is selected as a representative time series as it has been analysed in other works [1,2,15], and is known to be amenable to time series analysis. We hope to investigate other parameters in future work.

### 5.1. Univariate Analysis

**Figure 3:**
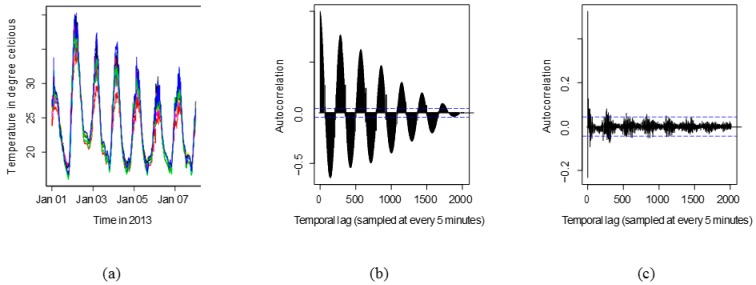
(**a**) Multiple time series plot for 12 nearby sensors; (**b**) Sample autocorrelation for a univariate temperature series; (**c**) Sample autocorrelation for differenced time series. Horizontal dashed lines indicate the ±5% bounds normally used to identify stationarity in the ACF.

Figure 3a shows the multiple time series plot of 12 nearby sensors superimposed. It helps to evaluate obvious inconsistencies among the series which is not present in this case. Figure 3b shows the temporal autocorrelation of temperature from one of the sensors. From the nature of the correlation, it is obvious that the series is non-stationary. Any series that possesses periodicity in their correlation are non-stationary. The Augmented Dicky-Fuller test is run for each time series to verify that its non-stationarity is of order 1. Also, the time series model identification utility available in R is used for model identification. Figure 3c shows that after first order differencing, the autocorrelation is reduced to small values for all lags, and so this differenced sequence is stationary, and amenable to analysis.

### 5.2. Co-Integration Analysis

After confirming that all series are first order non-stationary, co-integrated analysis is then performed for each node. The nodes are given ID’s ranging from node N1 to N12. Table 2 shows the statistics of the ADF test value for each sensor node with the rest of the nodes. 

In order for a series to be co-integrated with another, the test statistic should be less than the ADF test threshold which is normally set to −3.5, as described earlier in Section 3.1.4. It can be seen that almost all ADF test statistics are less than the critical value which means all series are statistically co-integrated. More negative values of the test statistic indicate a higher co-integration between series. Almost all series have a high degree of co-integration with all other series, with the test statistic for most pairs in Table 2 significantly more negative than the −3.5 threshhold. The exceptions are nodes 9 and 11 with a test statistic close to the threshold when paired with other series. Among the co-integrated series, some are highly co-integrated with a single series. Node N1, N3 and N5 are highly co-integrated with N2. Similarly, N4, N6 and N8 are most co-integrated with N7. N9 and N11 are less co-integrated with other nodes, but they are co-integrated with each other. Also, N10 and N12 are co-integrated with N1 which in turn is co-integrated with N2. Note that the most co-integrated node is rarely the physically Nearest Neighbour node, shown in the NN row in the table.

This co-integration result shows that three sensor nodes, namely N2, N7 and N11, are co-integrated with all of the rest of the nodes. This indicates that using these three co-integrated series, the remaining series should be able to be accurately estimated by using a linear estimator.

### 5.3. Estimation of Observation at Co-Integrated Nodes

This section analyses results about how co-integrated series can be used for the estimation of the temperature value. The best subset selection algorithm is used to search for the best subset of nodes among co-integrated nodes. The maximum subset to be selected is set to 1 to evaluate how useful the most co-integrated node is for the estimation of temperature at other sensor nodes. 

For each node, the most co-integrated node from Table 2 is selected as the estimator. Temperature is then estimated during a separate 10 day test period using the linear model learned during the training phase and mean test error is recorded.

**Figure 4:**
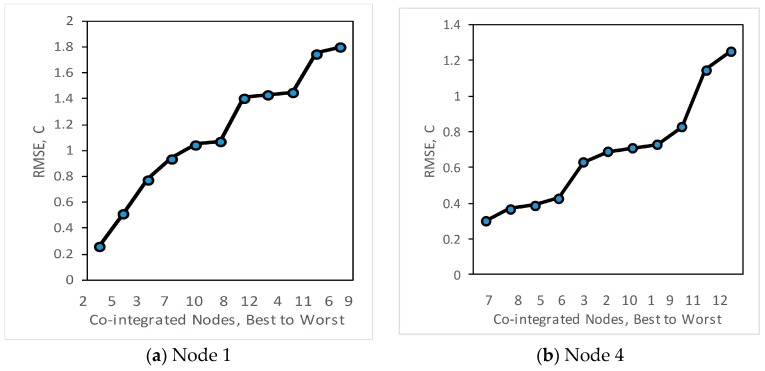

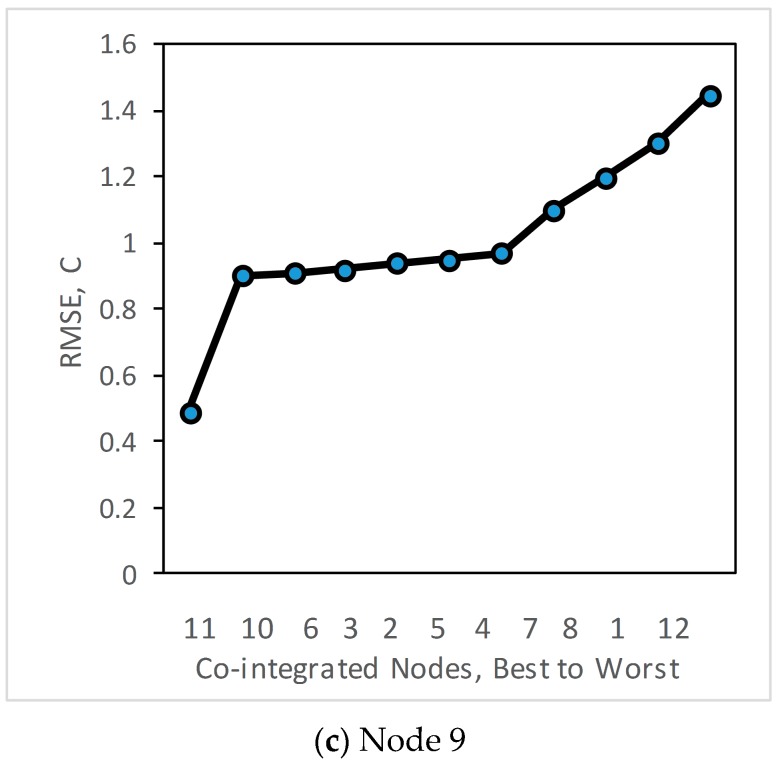
Root Mean-squared estimation error for co-integrated series at (**a**) Node 1, and (**b**) Node 4, and (**c**) Node 9, using all other nodes as estimators.

We then also analyse how the estimation varies if other nodes are selected instead of the most-co-integrated node. The RMSE is recorded for each of the other nodes used as an estimator. Figure 4 shows how the root mean squared error (RMSE) varies when different nodes are used for estimation — the order of nodes on the x-axis is from best to worst, left to right. The least RMSE for estimation of node N1 in Figure 4a is with the most co-integrated node N2 with an RMSE of 0.26 °C. 

Based on the ordering given by RMSE, the quality order (best to worst) of estimators is N2, N5, N3, N7, N10, N8, N12, N4, N6, N11, N9. It is worth noting that this is different to an ordering based on the ADF test statistic as shown in Table 2, where the most co-integrated nodes for N1 are (in order) N2, N3, N7, N10, N8, N5, N4, N6, N12, N9, N11. The ADF test statistic, as shown in Table 2, gives a measure of the confidence that two nodes are co-integrated, rather than a direct measure of the quality of prediction. So, we recommend using Algorithm 1, based on the ADF, to establish where nearby series are sufficiently co-integrated for this method to be valid, and then use algorithm 2 based on RMSE to actually select the best estimator nodes.

We repeat the analysis at node 4, which is most co-integrated with node 7 as shown in Figure 4b. From this figure it can be seen that RMSE for node 4 is small with mostly co-integrated nodes 7, 8, 5 and 6 while estimation error is higher with node 11 which is less co-integrated. In the case of node 9, the lowest RMSE is obtained with node 11 as shown in Figure 4c. 

If the RMSE error threshold for temperature measurement in all nodes were set to 0.5 °C, nodes 2, 7 and 11 would be sufficient to estimate all other nodes within the required accuracy. So the number of deployed nodes could be reduced by 75%.

**Figure 5:**
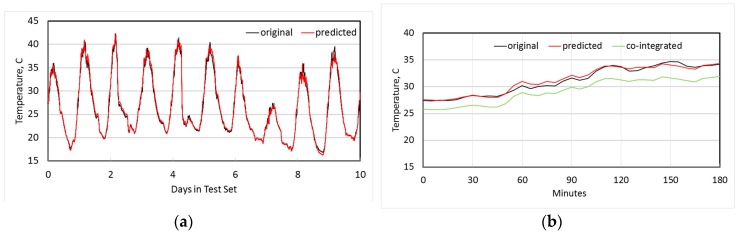
Estimation of temperature at node N1 using most co-integrated node N2 (**a**) over 10 days; (**b**) detail over first three hours, including the co-integrated baseline used for estimation.

**Figure 6:**
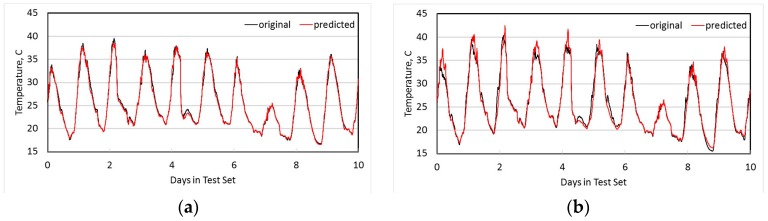
Estimation of temperature nodes N4 and N7. (**a**) N4 estimated from N7; (**b**) N9 estimated from N11.

Figure 5a shows both the original measured temperature at node N1, and the temperature estimated from using co-integrated node N2 over the 10 day test set. Figure 5b shows the detail of these two time series for the first 3 h, as well as the original measured temperature at N2, and it is clear that a linear estimator is significantly better than simply using N2 directly as an estimate. Figure 6a shows the original measured temperature at N4 and the estimated temperature from its most co-integrated node N7, while Figure 6b shows the original and estimated temperature at node N9. In all cases, the linear estimates from co-integrated nodes give good approximations to the actual measured temperatures. 

### 5.4. Discussion

While we have demonstrated the proposed approach on temperature time series, the approach is broadly applicable for determining the minimal set of sensor nodes for monitoring a given area. Since the sensor fields for each area will have unique spatiotemporal dynamics, our approach requires an initial dense deployment of sensor nodes for a short period. Once enough data is collected, we can determine nodes that are highly co-integrated and select the minimal set of nodes that can capture the sensor processes accurately. The deployment can then be reduced to include only the minimal set of nodes, thereby minimizing the monetary cost and network scale, along with its associated bandwidth overheads.

Several issues remain for future work. Firstly, how densely should the initial nodes be deployed? This obviously depends on the nature of the parameter being measured and its spatial variability. For this experiment, we have used temperature sensors that have been deployed at approximately 100 m intervals, and we have shown that 75% of sensors can be estimated by spatial interpolation. Our suggestion would therefore be to deploy sensors at approximately four times the density of the expected final deployment, with the expectation that 75% are unnecessary, but the remaining 25% will be placed at better positions. This is clearly an area for more future investigation.

**Figure 7:**
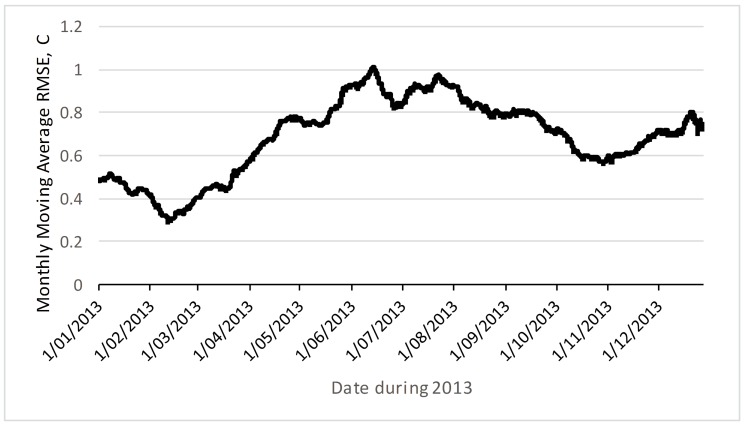
RMSE (moving average over 1 month) of prediction error using linear parameters from one week of training data in January.

A second question is whether the co-integrated prediction is reliable into the future, given that the test data in Figure 5 and Figure 6 is immediately after the training data. Figure 7 shows how the RMSE changes over the course of the next year, using estimation parameters from just one week of training data. The monthly moving average RMSE error peaks at about 1 °C in the opposite season (winter in July versus training data during summer in January). This suggests that the RMSE error in the opposite season may be twice that close to the training data. If the deployment is planned to be very long term, this suggests temporary deployments that includes summer and winter periods may be useful to get better prediction accuracy. Again, this is a fruitful area for further research. Another area for further research is the use of non-linear models, including more complex machine-learning estimators which could include the season as a prediction input.

## 6. Conclusions

This work has proposed a time series-based analytical approach to develop sampling node selection in environmental sensor networks. Co-integration is found to be a useful tool to investigate temporal variation of the monitored phenomena. From the analyses conducted with temperature series in a mine rehabilitation scenario, a significant number of sensing nodes are found to be redundant. Co-integrated nodes are shown to be capable of estimating observations at their co-integrated neighbour without exceeding a small error threshold. Such an approach of finding the best co-integrated nodes and using them to estimate observations for the rest of the nodes can be useful for developing a long term environmental monitoring strategy. 

To monitor a large spatial area, monitoring can begin with a large number of short-deployment sensors and analysing their co-integrated nature. Where sets of nodes are found to be co-integrated, redundant sensing positions can be removed. Permanent sensors are needed only in the positions of the non-redundant nodes. Alternatively, a small set of nodes can be densely deployed in one part of the area, the best positions chosen, then the unused nodes would be moved to another section of the area and this can be continued until the whole spatial region is covered. However, while this approach would provide local optima for sensor positions for each neighbourhood, it is more difficult to guarantee an optimum deployment over a large area. One suggestion would be to start at the centre of the deployment area, and then gradually move outwards. The pool of candidate nodes could include all the already committed permanent nodes from previous areas in the pool of potential co-integrated nodes. The best algorithm for extending this technique to cover a larger area would be an interesting topic for future work. 

Currently, this work only focuses on static sensor nodes. Future work could include using mobile nodes in to the monitoring to map the co-integrated regions of the sensing field prior to permanent node deployment.

## Figures and Tables

**Table 1:** Critical Values for Dickey-Fuller Test Statistic.

Sample Size	99% Confidence Level	95% Confidence Level
50	−3.58	−2.93
100	−3.51	−2.89
500	−3.44	−2.87
Infinity	−3.43	−2.86

**Table 2:** ADF-test for time series, Best Match **bold**, NN = Physically Nearest neighbour.

	N1	N2	N3	N4	N5	N6	N7	N8	N9	N10	N11	N12
N1	-	−43.26	−35.17	−25.90	−28.06	−24.65	−30.53	−29.79	−3.90	**−30.20**	−3.55	**−7.86**
N2	**−43.26**	-	**−45.02**	−28.53	**−29.82**	−26.89	−31	−30.33	−3.53	−27.60	−3.64	−7.02
N3	−35.18	**−45.01**	-	−25.36	−24.35	−25.21	−25.92	−25.08	−3.82	−26.42	−3.55	−6.58
N4	−26.07	−28.71	−25.19	-	−25.59	−29.65	−43.97	−42.87	−3.82	−29.49	−3.54	6.48
N5	−28.16	−29.91	−24.26	−25.67	-	−22.60	−24.43	−25.65	−3.91	−20.41	−3.57	−6.63
N6	−24.73	−26.96	−25.12	−29.75	−22.61	-	−30.01	−29.86	−3.84	−22.45	−3.56	−6.69
N7	−30.53	−31.13	−25.79	**−43.92**	−24.40	**−30.92**	-	**−49.12**	−3.83	−22.78	−3.57	−6.57
N8	−29.96	−30.48	−24.96	−42.05	−25.60	−29.90	**−49.09**	-	−3.87	−22.45	−3.56	−6.68
N9	−3.90	−3.93	−3.19	−3.16	−3.40	−3.31	−3.26	−3.37	-	−3.52	**−5.16**	−3.88
N10	−30.10	−27.49	−26.51	−20.69	−20.54	−22.59	−22.97	−22.29	−3.79	-	−3.57	−6.68
N11	−3.55	−3.55	−3.68	−3.74	−3.94	−3.98	−3.97	−3.02	**−5.13**	−3.44	-	−4.48
N12	−7.86	−7.07	−6.82	−6.77	−6.93	−7.01	−6.87	−7.02	−3.49	−7.25	−3.68	-
NN	N2	N4	N4	N2	N6	N5	N8	N7	N10	N9	N8	N10
Best	N2	N3	N2	N7	N2	N7	N8	N7	N11	N1	N9	N1
